# Extratympanic electrocochleography in the diagnosis of auditory neuropathy/auditory dyssynchrony

**DOI:** 10.1016/S1808-8694(15)30763-1

**Published:** 2015-10-19

**Authors:** Adriana Ribeiro Tavares Anastasio, Kátia de Freitas Alvarenga, Orozimbo Alves Costa Filho

**Affiliations:** 1 Doctor, Speech Therapist, Adjunct Professor, Speech Therapy Course, FMRP-USP; 2 Speech Therapist, Habilitation Degree (Livre-Docente) Associate Professor, Speech Therapy Course, FOB-USP; 3 Physician, Otologist, Full Professor, USP Bauru Campus

**Keywords:** audiometry evoked response, child, hearing loss, cochlear microphonic potentials

## Abstract

The brainstem auditory evoked potential (BAEP) is being extensively used as a method for the evaluation of cochlear function in individuals with diagnosis of auditory neuropathy/auditory dyssynchrony (AN/AD). In the absence of otoacoustic emissions, many cases of AN/AD have been diagnosed by the presence of CM identified in the BAEP.

**Aim:**

to demonstrate the clinical applicability of extratympanic electrocochleography (ET-Ecochg) in the differential diagnosis of AN/AD compared to the BAEP.

**Method:**

a 4-year-old child with a diagnosis of AN/AD seen at the Audiological Research Center was submitted to ET-Ecochg with a 2000 Hz tone burst in rarefaction and condensation polarities.

**Results:**

the ET-Ecochg exam was illustrated. Using an appropriate protocol, it was possible to demonstrate CM and to confirm it in the Ecochg, with a recording quality superior to that obtained in the BAEP.

**Conclusion:**

ET-Ecochg permitted a more detailed analysis of CM compared to the BAEP, thus showing clinical applicability for the investigation of cochlear function in AN/AD.

## INTRODUCTION

In the past 20 years, scientists have described patients with similar clinical findings, but with a wide variety of terms to name those symptoms, such as brainstem processing syndrome, central auditory dysfunction, neural synchrony disorder, auditory neuropathy, and recently auditory dyssynchrony.^1^^,^^2^

The term auditory neuropathy was first used in a paper published in 1996^3^ that attempted to classify patients that had a variety of auditory symptoms, a normal-functioning cochlea, and altered cochlear nerve function.

In 2001, some authors1 suggested the term auditory dyssynchrony instead of auditory neuropathy, as the cochlear nerve was not always injured, and therefore the old term would be semantically incorrect. Even with this recommendation, however, both terms are still used in papers that describe these patients. As a result, auditory neuropathy/dyssynchrony (AN/AD) has emerged as a third term.^3^, ^4^, ^5^, ^6^, ^7^, ^8^, ^9^, ^10^

AN/AD patients are subjects that may have normal tone thresholds or profound hearing loss. The condition is usually bilateral, but there are reports of unilateral cases in the literature. The disorder may be diagnosed at any age. Otoacoustic emissions (OAEs) and/or cochlear microphonics (CMs) are present regardless of the degree of hearing loss. Auditory brainstem evoked potentials (ABEPs), middle ear muscle reflexes and olivocochlear reflexes are absent. Audiograms, when they can be applied, may be flat or show an upward or downward-sloping curve or be completely irregular; findings usually do not match speech understanding difficulties.^1^^,^^11^, ^12^, ^13^, ^14^, ^15^

It may be difficult to locate the injury site in AN/AD, although the presence of OAEs and/or CMs suggests that outer hair cells (OHC) are intact. The ABEP wave I, however, which is generated by the myelin-sheathed peripheral portion of the cochlear nerve, is absent in this auditory condition. Symptoms suggest a disorder involving inner hair cells (IHC), the synapses between IHC and the auditory nerve, and peripheral portions of the cochlear nerve.^16^, ^17^, ^18^

Although the risk factors for AN/AD are unclear, some of the children with this disorder have a history of neonatal events such as premature birth, low birth weight, anoxia and hyperbilirubinemia.^19^

The presence of OAEs associated with absent ABEPs is pathognomonic of AN/AD, although in some cases OAE recordings may be absent. Consequently, depending on when the initial diagnosis is made, OAEs and absent ABEPs will not provide a final definition and diagnosis of AN/AD. In such cases, the presence of CMs becomes the determinant finding in the differential diagnosis of this condition.^1^^,^^20^^,^^21^ Some authors have proposed that AN/AD be defined as a condition with absent or abnormal ABEPs including wave I, associated with present OAEs and/or CMs.^20^

In the past 10 years, many papers on auditory neuropathy have underlined the role of CMs, which are now analyzed with greater care in AN/AD patients. Reports in the literature have shown that CMs in these patients are more prominent, having an abnormally increased amplitude,^23^, ^24^, ^25^, ^26^ and persist for up to 4 to 6 milliseconds after click stimulation,^22^ contrary to what is found in normal subjects.

Electrocochleography (ECoG), which had a significant clinical impact in the diagnosis and monitoring of Ménière's disease,^27^, ^28^, ^29^ and which objectively assesses cochlear potentials, is the indicated clinical procedure to analyze CMs.^30^^,^^31^ The recent development of various non-invasive electrodes have reawakened interest in the clinical use of ECoG.^27^^,^^32^ Although transtympanic electrocochleography (TT-ECoG) yields recordings with higher amplitudes and lower test-retest variability, it has the disadvantage of being an invasive procedure. Extratympanic electrocochleography (ET-ECoG), therefore, is clinically more useful in this context, supporting an audiological diagnosis and increasing knowledge about cochlear function in AN/AD.

Even though ECoG is the most appropriate procedure for assessing cochlear function, and therefore helping identify CMs and diagnosing AN/AD, there is little information in the literature on its use in this disorder.^4^^,^^5^^,^^33^

ABEP testing has also been widely used in identifying CMs, the rationale being that insertion phones avoid the extensive magnetic field caused by supra-aural phones, which obscure cochlear potentials and, at times, the nerve action potential. Furthermore, CMs may be identified by recording auditory evoked potentials using negative and positive polarity acoustic stimuli in which results may be compared, given that CMs are affected by the stimulus polarity, contrary to ABEPs.^1^^,^^20^^,^^25^^,^^34^

To confirm the presence of CMs, some researchers block the insertion phone plastic tube to guarantee that recordings are auditory neurophysiological responses that truly reflect acoustic stimulation, rather than electrical artifacts.^3^^,^^20^^,^^24^

Although the literature has suggested that cochlear function is normal in AN/AD,^3^^,^^13^^,^^34^ it is important to analyze CMs in these cases; a number of findings about this specific cochlear potential in AN/AD patients appear to show that the cochlea may be dysfunctional.^23^, ^24^, ^25^, ^26^

The use of ABEP testing of CMs seems inadequate, as CMs cannot be carefully analyzed by this method. A better analysis of CM amplitude and morphology would be obtained with tone bursts, rather than the clicks usually used in ABEP testing.^28^^,^^35^^,^^36^

Based on this assumption, the aim of this paper was to demonstrate the clinical applicability of ET-ECoG in the differential diagnosis of AN/AD, compared to ABEP testing.

## MATERIAL AND METHOD

A case description is made of a four-year-old child diagnosed with AN/AD. Following a term birth and the presentation of apnea and cyanosis three days later, the child was admitted into hospital and given gentamycin for 14 days. Hyperbilirubinemia ensued, requiring blood transfusion. Free-field audiological testing found a decreased auditory threshold that suggested a downward-sloping configuration moderate to severe hearing loss. Responses to intense speech were inconsistent. Immitance testing showed normal tympanometric curves and absent contralateral and ipsilateral acoustic reflexes. Transient and distortion product OAEs were present in both ears.

ABEP testing and ET-ECoG were done to aid in the differential diagnosis of AN/AD. The test protocol is described in Table 1. Both ABEP testing and ET-ECoG were done in an acoustically and electromagnetically treated room, using a Bio-logic Systems Corp.® Navigator Pro device, version 4.2.0. The skin was cleaned with abrasive paste; a small amount of electrolytic paste was placed on disposable electrodes. After electrode placement, the cables were connected. An Ear Tone 3A earphone was used. The following electrode arrangement was used for ABEP testing: active-frontal; reference-mastoid, and ground on the contralateral mastoid to the stimulated side. The active and ground ET-ECoG electrodes were placed frontally, and the reference electrode (TIPtrode model) was introduced into the acoustic canal.

**Table 1:** Protocol for ABEP testing and ET-ECoG recording.

Parameters	ABEP	ET-ECoG
Type of stimulus	click	Tone burst − 2,000Hz
Stimulus velocity	21.1	19.30
Stimulus intensity	90dBnHL	80dBnHL
Stimulus polarity	Rarefaction/condensation	Rarefaction/condensation
Stimulus duration	100μs	1–3–1 (linear)
Promediated stimulus	2000 replicated	500 replicated
Masking	No	No
Type of earphone	Insertion	Insertion
Analysis time	15ms	10.66ms
High-pass filter	100Hz	30Hz
Low-pass filter	3000Hz	5000Hz
Amplification (gain)	–	75.000
Artifact rejection limit	Up to 10% of the total	Up to 10% of the total
Pre-stimulus interval	–	1ms

Etymotic Research laboratories, based on insertion phone structure, developed the TIPtrode. It contains a plastic tube that conveys sound and that connects to the insertion phone tube. It is coated by a thin gold layer that conducts the auditory evoked potential electrical activity to the preamplifier.^37^ The TIPtrode not only initiates acoustic stimuli, but is also the receptor for electrical activity. Following the registration protocol, the procedure for both ABEP testing and ET-ECoG was repeated in rarefaction polarity by blocking the insertion phone plastic tube with appropriate forceps that had two rubber olives on its extremity to avoid damage to the plastic tube and to stop the acoustic stimulus from reaching the auditory canal. In these circumstances, the cochlear potential disappears, and only any signal electrical artifacts remain.^20^^,^^24^^,38^ This sequence aimed to assure the reliability of recordings and to confirm the biological response.

## RESULTS

**Figure 1:**
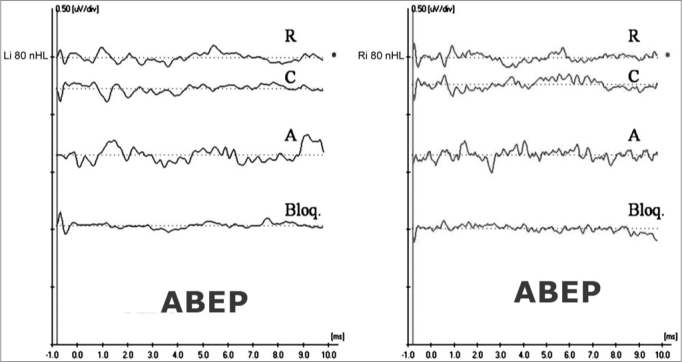
Extratympanic electrocochleography in auditory neuropathy/dyssynchrony - ABEP recordings in AN/AD

Figure 1 shows ABEP recordings in both ears in a four-year-old child diagnosed with AN/AD. CMs may be seen in the two upper recordings (R-rarefaction, and C-condensation) as well as absence of neural components. Cochlear CMs were not completely cancelled in the intermediate recording (A-alternated). The lower recording (Bloq-blocked) shows the absence of a biological response.

**Figure 2:**
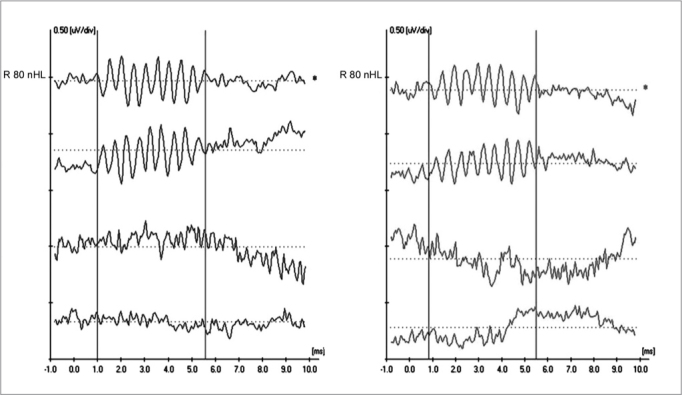
Extratympanic electrocochleography in auditory neuropathy/dyssynchrony - ET-ECoG recordings in AN/AD

Figure 2 shows ET-ECoG recordings in both ears in a four-year-old child diagnosed with AN/AD. CMs may be seen in the two upper recordings (R-rarefaction, and C-condensation) as well as absence of neural components. Cochlear CMs were not completely cancelled in the intermediate recording (A-alternated). The lower recording (Bloq-blocked) shows the absence of a biological response. CMs are seen with better quality in these recordings, making it possible to analyze CM morphology, amplitude and duration more precisely.

**Figure 3:**
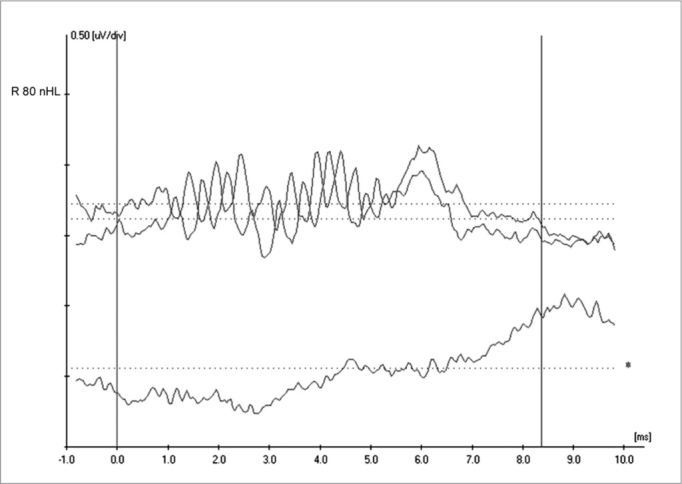
Extratympanic electrocochleography in auditory neuropathy/dyssynchrony - Inversion of CM tracings in ET-ECoG

Figure 3 shows inversion of CM tracings at 180° on the upper ET-ECoG recording when using rarefaction (R) e condensation (C) polarities. CM authenticity is shown on the lower recording, where the cochlear potential disappears when blocking the insertion phone tube (Bloq-blocked).

## DISCUSSION

Auditory neuropathy/dyssynchrony (AN/AD) is a relatively new condition in audiology that has been investigated in many recent papers. OAEs and ABEP testings are the methods used in diagnosing this disorder. When OAEs are absent in these patients, however, the differential diagnosis depends on CM recordings.^1^, ^2^, ^3^, ^4^, ^5^, ^6^, ^7^, ^8^, ^9^, ^10^, ^11^, ^12^, ^13^, ^14^, ^15^, ^16^, ^17^, ^18^^,^^20^^,^^21^

It seems inappropriate to analyze CMs by using ABEP testing for the following reasons: the stimulus (click) that is used to initiate neural potentials limits the conditions for recording CMs;^1^^,^^20^^,^^25^^,^^34^ and secondly, many clinicians perform ABEP testing with supra-aural phones, in which blocking the plastic tube to confirm a biological response is not possible.

ET-ECoG is a non-invasive technique that may be used in young children, without sedation; the diagnosis of AN/AD, therefore, may be expedited. The recording amplitude is increased, compared to ABEP testing, by locating the electrodes closer to the site of origin of cochlear potentials, which is extremely relevant in AN/AD, as published papers have shown that CMs are ampler in such testing conditions.^22^, ^23^, ^24^, ^25^, ^26^ Higher quality recordings make it possible to evaluate not only the amplitude but also the duration of CMs; the literature suggests that CMs are more prolonged in AN/AD, compared to normal subjects.

A further points is that ET-ECoG also evaluates cochlear function, thereby providing an audiological assessment if OAEs are absent.

It is important to note that even when using ET-ECoG to evaluate CMs in AN/AD patients, an appropriate protocol using strategies to assure recording reliability should be applied. Tone burst stimulation should be used, and rarefaction and condensation polarities should be used to confirm inversion of recordings, and therefore, confirmation of CMs. We also recommend using insertion phones to provide confirmation of a biological response, where the plastic tube may be blocked to discard possible signal electrical artifacts.

## CONCLUSION

Our findings show that ET-ECoG may be applied clinically in the differential diagnosis of AN/AD, making it possible to analyze cochlear function in greater detail compared to ABEP testing, particularly in identifying CMs.

We aimed to provide additional data in support of a more effective diagnosis of AN/AD, especially in small children in whom electrophysiological methods frequently are the only available tools for evaluating auditory function.
